# Post-COVID-19 condition and persisting symptoms in English schoolchildren: repeated surveys to March 2022

**DOI:** 10.1186/s12879-023-08203-1

**Published:** 2023-04-05

**Authors:** Charlotte Warren-Gash, Andrea Lacey, Sarah Cook, Dylan Stocker, Samantha Toon, Ffion Lelii, Ben Ford, Georgina Ireland, Shamez N. Ladhani, Terence Stephenson, Patrick Nguipdop-Djomo, Punam Mangtani, the COVID-19 Schools Infection Survey 2 Study Group, Elliot McClenahan, Gillian McKay, Jody Phelan, Liang-Yu Lin, Alex Lewin, Alison Judd, Byron Davies, Anisah Saib, James McCrae, Joe Kelly, Ian Diamond, Emma Rourke, Fiona Dawe, Pete Jones

**Affiliations:** 1 Department of Non-Communicable Disease Epidemiology, Faculty of Epidemiology and Population Health, London School of Hygiene & Tropical Medicine, Keppel Street, WC1E 7HT London, UK; 2 Office for National Statistics, Government Buildings, Newport, UK; 3 School of Public Health, Imperial College London, London, UK; 4 Public Health Programmes, UK Health Security Agency, London, UK; 5 UCL Great Ormond Street Institute of Child Health, London, UK; 6 Department of Infectious Disease Epidemiology, Faculty of Epidemiology and Population Health, London School of Hygiene & Tropical Medicine, London, UK

**Keywords:** Schools, Children and young people, England, Post-COVID-19 condition, Persisting symptoms

## Abstract

**Background:**

Both post-COVID-19 condition (long COVID) and the presence of persisting symptoms that do not meet formal definitions of post-COVID-19-condition may adversely affect quality of life and function. However, their prevalence among children and young people in England is unclear.

**Methods:**

We used data from repeated surveys in a large cohort of English schoolchildren from the COVID-19 Schools Infection Survey (SIS) for the school year 2021/22 to describe the weighted prevalence of post-COVID-19-condition and compare persisting symptoms between individuals with a positive SARS-CoV-2 test and those with neither a positive test history nor suspected infection.

**Results:**

Among 7797 children from 173 schools, 1.8% of primary school pupils (aged 4 to 11 years), 4.5% of secondary school pupils in years 7–11 (aged 11 to 16 years) and 6.9% of those in years 12–13 (aged 16 to 18 years) met a definition of post-COVID-19 condition in March 2022. Specific persisting symptoms such as anxiety or difficulty concentrating were frequently reported regardless of prior infection status and increased with age: 48.0% of primary school pupils, 52.9% of secondary school pupils in years 7–11 and 79.5% in years 12–13 reporting at least one symptom lasting more than 12 weeks. Persisting loss of smell and taste, cardiovascular and some systemic symptoms were more frequently reported by those with a previous positive test.

**Conclusions:**

We showed that ongoing symptoms were frequently reported by English schoolchildren regardless of SARS-CoV-2 test results and some specific symptoms such as loss of smell and taste were more prevalent in those with a positive test history. Our study emphasises the wide-ranging impacts of the COVID-19 pandemic on the health and wellbeing of children and young people.

**Supplementary Information:**

The online version contains supplementary material available at 10.1186/s12879-023-08203-1.

## Background

While COVID-19, the clinical disease associated with acute SARS-CoV-2 infection, is usually of short duration and low symptom burden in children and young people (CYP) [1], persisting physical and psychological symptoms are frequently reported after infection [2]. Post COVID-19 condition (long COVID) among CYP has been defined through a modified DELPHI process as ‘*a history of confirmed SARS-CoV-2 infection, with at least one persisting physical symptom for a minimum duration of 12 weeks after initial testing that cannot be explained by an alternative diagnosis. The symptoms have an impact on everyday functioning, may continue or develop after … infection, and may fluctuate or relapse over time*’ [3]. While more detailed, this research definition of long COVID is in accordance with clinical case definitions developed by the National Institute for Health and Care Excellence [4].

During the COVID-19 pandemic, however, many CYP who have tested negative for SARS-CoV-2 also report persisting symptoms such as fatigue as well as feelings of worry or unhappiness [5]. This may partly reflect disruptions to schools and social connectedness during the pandemic [6]. Comparing the prevalence of persisting symptoms among CYP with and without confirmed or probable COVID-19 across studies is challenging due to varying case definitions, study designs, lengths of follow up and symptom groupings [7]. Nevertheless, understanding the burden of persisting symptoms in these groups is essential to guide strategies to mitigate the impact of COVID-19 on CYP.

Here we aimed to (i) describe the prevalence of post-COVID-19 condition (long COVID) [3] and (ii) describe the prevalence and distribution of persisting specific symptoms [8] among CYP reporting a previous positive SARS-CoV-2 test compared to those with neither a positive test nor previous suspected infection using data from a representative sample of English schools collected through the COVID-19 Schools Infection Survey.

## Methods

We used data from the COVID-19 Schools Infection Survey (SIS) for the school year 2021/22. The original Schools Infection Survey was set up in the school year 2020/21 to monitor the prevalence of SARS-CoV-2 in schools in England and to investigate risk and protective factors for transmission [9]. In 2021/2, the scope broadened to include a focus on physical and mental health during the pandemic. Our study collected health information through three survey rounds (round one: Nov-Dec 2021; round two: Jan-Feb 2022 and round three: Mar-Apr 2022) in a nationally representative cohort of pupils from a stratified random sample of primary and secondary schools in 42 local authorities (LAs) in England. Sampling procedures are described elsewhere [10]. In brief, we planned to enrol 13 primary and 7 secondary schools in each of England’s nine geographic regions from October 2021 aiming to reach a target of 180 schools. This target was reached through sample size calculations based on a primary outcome of antibody prevalence which are reported elsewhere [11]. All pupils aged 4 years and over were invited to participate.

Online consent forms and questionnaires were completed by parents/guardians for children in school years 0–11 (aged 4–16 years) and by pupils in school years 12–13 (aged 16–18 years). Other study procedures are described elsewhere [10]. For this study, data for pupils in school years 7–11 (aged 11–16 years) were taken from parent questionnaires only. Questionnaires captured self-reported information on household composition, socio-demographics, medical and symptom history, SARS-CoV-2 test results, social contacts and mental wellbeing [10]. History of confirmed SARS-CoV-2 infection was defined as self-reporting a positive result on either PCR or lateral flow testing since March 2020. Suspected COVID-19 was based on a positive response to the question ‘Do you know or think that [child’s first name] has had coronavirus (COVID-19) since March 2020?’ We also collected data on the presence, features and impact of symptoms experienced in a persisting or recurring way for more than 12 weeks since March 2020. The wording of the questions used to generate outcome and comparator definitions is shown in the Supplementary methods.

Questionnaire responses were captured in round one (before Omicron) and in round three (during Omicron subvariant waves) when additional participants had been recruited. The dates for round one were 22^nd^ November-15^th^ December for parent questionnaires, with pupils in years 12–13 given five weeks to respond (11^th^ November-15^th^ December) to maximise uptake. For round three, questionnaire delivery was streamlined to a two-week period (15^th^ March-1^st^ April) for both parents and young people in years 12–13 for administrative reasons. Round two did not include questions on long COVID.

Using these data, we first described the weighted prevalence (%) of post-COVID-19-condition (long COVID) meeting a definition based on the DELPHI consensus [3]. This was reporting of a positive SARS-CoV-2 test*,* the presence of symptoms continuously for 12 weeks or more after the start of their infection, and everyday life being affected by those symptoms. Data were described by school type, age-group, sex, eligibility for free school meals (government-funded school meals for socio-economically disadvantaged children; self-reported at registration) and presence of underlying health conditions. Second, we described the weighted % of all CYP experiencing persisting symptoms lasting more than 12 weeks since March 2020 by age group. We explored persisting symptoms both individually and grouped according to an approach taken by the Patient-Led Research Collaborative [8]. For each symptom and grouping, we calculated differences between those reporting a previous positive SARS-CoV-2 test and those with neither a history of a positive test nor suspected COVID-19 for each age group, generating exact binomial 95% confidence intervals for difference in proportions [12].

Ethical approval was obtained from the UKHSA Research Support and Governance Office (R&D 474) and London School of Hygiene & Tropical Medicine Ethics Review Committee (ref:22,657).

## Results

One hundred thirty-four schools participated in round one, with questionnaire data available from 3,375 parents for 4,128 children in school years 0–11 (aged 4–16 years) and from 143 pupils in years 12–13 (aged 16–18 years). In round three, there were 173 registered schools, with questionnaire data available from 6,197 parents for 7,448 children in years 0–11 and from 349 pupils in years 12–13.

In round one, 1% (0.5 to 1.6%) of 2,485 primary school pupils surveyed, 2.5% (1.4 to 4.2%) of 1,902 pupils in years 7–11 and 4.4% (1.1 to 11.6%) of 143 pupils in years 12–13 were reported to have experienced long COVID as a result of their most recent SARS-CoV-2 infection. In round three, the prevalence of long COVID was 1.8% (1.2 to 2.6%) among 3,788 primary school pupils, 4.5% (3.4 to 5.7%) among 2,617 pupils in years 7–11 and 6.9% (3.3 to 12.4%) for 349 pupils in years 12–13, respectively. No clear patterns were seen by gender, the presence of underlying health conditions or eligibility for free school meals, a measure of socio-economic deprivation (Table 1).

**Table 1:** Weighted prevalence of long COVID meeting a definition based on the DELPHI consensus definition in survey rounds one and three by pupil characteristics

Age-group	Characteristic	Round one (Nov/Dec 2021)		Round three (Mar/Apr 2022)	
		Unweighted denominator	Weighted %^(a)^ with long COVID^(b)^ (95% C.I.)	Unweighted denominator	Weighted %^(a)^ with long COVID^(b)^ (95% C.I.)
Primary school years 0–6 (aged 4–11 years)	Overall	2,485	1.0 (0.5 to 1.6)	3,788	1.8 (1.2 to 2.6)
	Gender				
	Female	1,227	1.5 (0.8 to 2.7)	1,880	2.7 (1.7 to 4.0)
	Male	1,258	0.4 (0.1 to 1.2)	1,908	1.0 (0.4 to 2.0)
	Underlying health conditions^c^				
	Present	234	1.2 (0.1 to 1.8)	319	3.8 (1.3 to 8.2)
	Absent	2,216	1.0 (0.5 to 1.7)	2,868	1.5 (0.9 to 2.4)
	Missing	35		601	
	Free school meals				
	Eligible	518	1.1 (0.3 to 3.0)	855	1.3 (0.4 to 3.0)
	Not eligible	1,848	0.8 (0.4 to 1.6)	2,748	2.0 (1.3 to 2.9)
	Missing	119		185	
Secondary school years 7–13 (aged 11 to 18 years)	Overall	2,045	2.7 (1.6 to 4.3)	2,966	4.8 (3.7 to 6.0)
	Age-group				
	Years 7–11	1,902	2.5 (1.4 to 4.2)	2,617	4.5 (3.4 to 5.7)
	Years 12–13	143	4.4 (1.1 to 11.6)	349	6.9 (3.3 to 12.4)
	Gender				
	Female	1,070	2.2 (0.9 to 4.5)	1,641	5.8 (4.2 to 7.7)
	Male	975	3.2 (1.6 to 5.7)	1,325	3.8 (2.5 to 5.5)
	Underlying health conditions				
	Present	168	5.6 (0.7 to 19.1)	330	4.8 (2.0 to 9.5)
	Absent	1,074	1.7 (0.6 to 3.8)	2,276	4.4 (3.3 to 5.8)
	Missing	803		360	
	Free school meals				
	Eligible	194	2.3 (0.1 to 9.8)	284	5.3 (2.1 to 10.8)
	Not eligible	1,809	2.7 (1.5 to 4.4)	2,615	4.6 (3.6 to 5.9)
	Missing	42		67	

^a ^Estimates are weighted for sampling design, school-level response rate, school years, sex, ethnicity and free school meal eligibility (a measure of socio-economic deprivation)

^b^ Long COVID defined as self-reporting a positive SARS-CoV-2 test (PCR or lateral flow) since March 2020*,* the presence of symptoms continuously for ≥ 12 weeks and everyday life being affected by symptoms

^c^ Underlying health conditions included cancer, diabetes, serious heart problems, chest complaints or breathing difficulties including poorly-controlled asthma, kidney, liver or a gut disease, lowered immunity due to disease or treatment (such as steroid medication, chemotherapy or radiotherapy), an organ transplant, a neurodisability or neuromuscular condition, a severe or profound learning disability, Downs syndrome, a problem with the spleen e.g. sickle cell disease or spleen removal, epilepsy, serious genetic problems, serious allergies e.g. eczema, allergic rhinitis or food allergies, other serious medical conditions as advised by a doctor or specialist

A history of symptoms persisting for more than 12 weeks was common regardless of infection history, with 48.0% (45.6 to 50.5%) of primary school pupils, 52.9% (50.5 to 55.3%) of secondary school pupils in years 7–11 and 79.5% (71.9 to 85.8%) of those in years 12–13 reporting at least one persisting symptom in round three. Similar results were reported in round one (Supplementary table 1). For primary school pupils, the most common persisting symptoms in both rounds were cough, sore throat, worry or anxiety and difficulty concentrating. In secondary schools in both rounds, worry or anxiety was the most frequently reported symptom, with low mood and difficulty concentrating both in the commonest four symptoms, as well as sore throat (years 7–11) and weakness or tiredness (years 12–13) (Supplementary table 1).

**Fig. 1:**
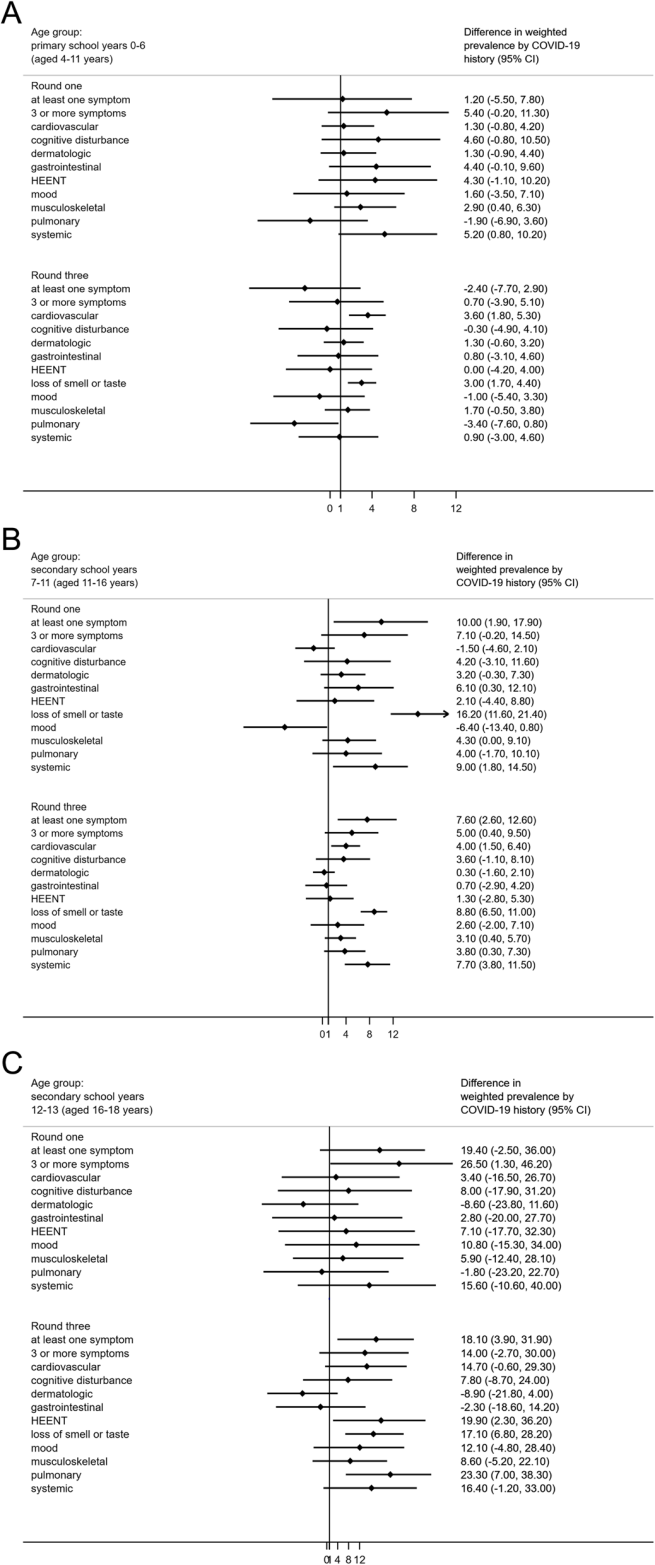
Difference in weighted^1^ prevalence of grouped symptoms^2^ reported between individuals with a history of a positive test and those with neither a positive test nor suspected COVID-19 in rounds one^*^ and three^±^, by age group (**A**: primary school years 0–6; **B**: secondary school years 7–11; **C**: secondary school years 12–13). ^1^ Estimates are weighted for sampling design, school-level response rate, school years, sex, ethnicity and free school meal eligibility (a measure of socio-economic deprivation). ^2^ Grouped symptoms comprised the following: cardiovascular (chest pain or tightness; palpitations), cognitive disturbance (feeling lightheaded or disorientated; feeling dizzy; trouble sleeping; headache; memory loss or confusion; difficulty concentrating), dermatologic (raised, red, itchy bumps on skin or swelling of face or lips; red or purple blisters on feet or toes; prickling, tingling or burning sensations in skin), gastrointestinal (diarrhoea; not feeling hungry or wanting to eat; feeing or being sick; stomach pain), HEENT (lost or husky voice; sore throat; sore or uncomfortable eyes; earache or ringing in ears), loss of smell or taste, mood (worry or anxiety; low mood or not enjoying anything), musculoskeletal (strong aches or pains in joints or muscles), pulmonary (cough; feeling short of breath), systemic (fever or high temperature; chills or shivers; weakness or tiredness). ^*^ In round one, 532 primary school pupils had a history of a positive test compared to 1,791 with no reported COVID. For years 7-11, 515 pupils reported a positive test history compared to 1,037 with no reported COVID. The corresponding figures for years 12-13 were 39 and 90. ^±^ In round three, 2,130 primary school pupils had a history of a positive test compared to 1,334 with no reported COVID. The corresponding figures were 2,027 and 1,415 for pupils in years 7-11 and 161 and 159 for pupils in years 12-13. Note that in round one, figures for loss of smell and taste for primary school years 0-6 and secondary school years 12-13 were suppressed due to small numbers

In round three, 2,027 secondary school pupils in years 7–11 reported a previous positive test, 216 had had suspected COVID while 1,415 had neither a history of a positive test nor suspected COVID-19 (weighted percentages 54.0%, 6.8% and 39.2% respectively). More secondary school-aged pupils reported at least one persisting symptom if they had had a positive SARS-CoV-2 test (55.4% (52.2 to 58.6%)) compared to those with neither a history of a positive test nor suspected COVID-19 (47.8% (43.9 to 51.6%)). However, among primary school pupils, of whom 2,130 reported a previous positive test, 319 had had suspected COVID-19 and 1,334 had no positive test or suspected COVID-19 (weighted percentages 53.0%, 8.9%, 38.1%), there were no differences in those reporting at least one persisting symptom by COVID-19 history (Fig. 1; Supplementary table 2).

For individual symptoms, the most striking difference was for loss of smell or taste, which was consistently reported more frequently by pupils with a positive test compared to those with neither a positive test nor suspected COVID-19 in all age groups in round three (3.1% versus 0.1% in years 0–6, 10.3% versus 1.5% in years 7–11, 18.4% versus 1.3% in years 12–13). Cardiovascular symptoms (chest pain or tightness, palpitations) were also more commonly reported among children in school years 0–11 with a positive test than those with neither a positive test nor suspected COVID-19: 4.8% v. 1.2% in primary school and 8.5% v. 4.5% for pupils in years 7–11. For those in years 7–11, systemic symptoms (fever, chills, weakness or tiredness) were more frequently reported among those with a positive test: 22.1% v. 14.4%. For years 12–13, while numbers were small, pulmonary symptoms and symptoms affecting the head, eyes, ears, nose and throat were also more prevalent among those with a positive test (Fig. 1; Supplementary table 2).

## Discussion

In this large cohort of schoolchildren in England, the prevalence of post-COVID-19 condition (long COVID) in March 2022 was low, ranging from 1.8% in primary school pupils to 6.9% among those in school years 12–13. Specific persisting symptoms such as worry or anxiety and difficulty concentrating were frequently reported regardless of prior infection status and increased with age. Persisting loss of smell and taste along with cardiovascular and some systemic symptoms showed the most marked difference in prevalence by infection history.

The low prevalence of cases meeting a definition of post-COVID-19 condition (long COVID) based on the DELPHI consensus definition is compatible with estimates from other studies of community cases in CYP [13] and lower than estimates from studies of hospitalised cases [14]. Similarly, the high prevalence of reported specific persisting symptoms is also consistent with other studies. A systematic review and meta-analysis of 17 studies of children with confirmed or probable COVID-19 with a median follow up of 125 days, showed pooled prevalence estimates for persisting symptoms ranging from 15% (diarrhoea) to 47% (fatigue) [2]. As in our study, others have shown relatively small differences in symptom reporting when comparing infected and uninfected groups: among 6 studies comparing persisting symptoms between children with and without a history of COVID-19, the difference in the proportion reporting persisting specific symptoms varied from -0.5% to 13.2% between groups (median difference 3.0%, IQR 1.4 to 3.6%) [7]. Nevertheless, these differences may increase with age: as in our study, a Danish community-based study of adolescents aged 15 to 18 years showed that those with a positive test history were more likely to report at least one persisting symptom than a matched control group [15].

These results highlight the high proportion of CYP reporting persisting symptoms during the pandemic, despite not meeting definitions of post-COVID-19 condition (long COVID). Repeated representative longitudinal surveys conducted in the UK also show that rates of probable mental disorders among CYP have increased steeply from 2017 to 2020–21 [16]. Reasons for this, including any impacts of the seasonality of survey administration on symptom reporting, are unclear. However, COVID-19 mitigation measures to reduce risk of infection by limiting physical access to schools, childcare, social activities, as well as health and social care services may have contributed to negative outcomes for the health and wellbeing of CYP. Measures to support the mental health and wellbeing of CYP regardless of prior infection status, including recognising the importance of school and family connectedness [6], should be a priority.

Strengths of our study include its large size, broad geographic representation across England and the use of repeated measures of persisting symptoms over time among CYP with and without a reported history of COVID-19. Nevertheless, the self-reported nature of symptoms lasting for more than 12 weeks and proxy reporting used for younger children may have led to some misclassification of persisting symptoms. In addition, the time period between initial infection and survey completion was variable with a maximum gap of two years for any child infected in March 2020 completing surveys in round three, which could lead to symptom misclassification. The limited data on timing of initial infection did not allow us to assess the impact of any time lag on symptom reporting. We also did not have data on repeated infections or information about whether an infection led to hospitalisation, which might have affected reporting of persisting symptoms. This could be an area for future research.

Our comparison group of those with neither a history of positive test nor suspected infection might include pupils infected but not diagnosed before the introduction of mass community testing around June 2020, though we tried to minimise the bias by excluding those with suspected (likely symptomatic) infection. We also cannot exclude selection bias among those who both agree to participate and chose to complete questionnaires. Another limitation is that the study only covers state-funded primary and secondary schools and excludes others such as independent and special schools, although it is estimated that 93% of pupils attend state funded schools [17]. Finally, numbers for some stratified analyses, particularly in the oldest age group were small.

## Conclusions

The increasing, albeit low, prevalence of post-COVID-19-condition (long COVID) among CYP and the high proportion reporting persisting symptoms regardless of COVID-19 test results, emphasise the wide-ranging impacts that the COVID-19 pandemic has had on CYP. Further work is needed to understand how persisting symptoms affect educational outcomes and quality of life longer-term. Emerging from the pandemic, child health and wellbeing must be prioritised to avoid embedding long-term disadvantage for a generation of CYP.

## Supplementary Information

**Additional file 1.**

## Data Availability

The datasets supporting the conclusions of this article are available on the Office for National Statistics website at the following URLs: https://www.ons.gov.uk/peoplepopulationandcommunity/healthandsocialcare/conditionsanddiseases/datasets/covid19schoolsinfectionsurveyquestionnairedataengland  and https://www.ons.gov.uk/peoplepopulationandcommunity/healthandsocialcare/conditionsanddiseases/adhocs/14808covid19schoolsinfectionsurveyenglandsymptomprevalencebycovid19statusnovembertodecember2021
