# Supplementary material for: Post-COVID-19 condition and persisting symptoms in English schoolchildren: repeated surveys to March 2022
Source: BMC Infect Dis. 2023 Apr 5;23:201. doi: 10.1186/s12879-023-08203-1 (PMC10075149; doi:10.1186/s12879-023-08203-1)
Supplement: Supplementary file 1 — Additional file 1. [file 12879_2023_8203_MOESM1_ESM.docx]

**Post-COVID-19 condition and persisting symptoms reported in English schoolchildren: March 2020 - March 2022: Supplementary material**

**Contents**

[**Supplementary methods** 2](#_Toc130223084)

[**Outcome definitions** 2](#_Toc130223085)

[**Comparator definitions** 4](#_Toc130223086)

[**Supplementary results** 5](#_Toc130223087)

[**Supplementary table 1** Prevalence of individual symptoms persisting for at least 12 weeks since March 2020 regardless of COVID-19 history, by age-group in survey rounds one and three 5](#_Toc130223088)

[**Supplementary table 2** Persisting symptom prevalence by COVID-19 status and age-group in survey rounds one and three 8](#_Toc130223089)

# **Supplementary methods**

## **Outcome definitions**

(i) The outcome of post-COVID-19-condition (long COVID) was defined based on responses to the following questions:

1. Have you / your child had a positive coronavirus (COVID-19) test of any kind since March 2020?
2. Have you / your child had at least one symptom of coronavirus (COVID-19) that continued for 12 weeks or more after the start of your coronavirus (COVID-19) infection?'
3. How much have the ongoing coronavirus (COVID-19) symptoms affected the following for you/ your child?

| Domains | Not affected | Affected a little | Affected a lot | Prefer not to say |
| --- | --- | --- | --- | --- |
| Your ability to learn |  |  |  |  |
| Your ability to stay physically active |  |  |  |  |
| Your emotional wellbeing |  |  |  |  |
| Your ability to carry out day to day activities |  |  |  |  |

To meet this definition, a positive response was required to questions 1 and 2, along with a statement that any of the domains had been affected either a little or a lot by coronavirus symptoms

(ii) Persisting symptoms were captured by positive responses to the following question:

1. Regardless of whether they have had coronavirus (COVID-19), which of the following symptoms has [child’s first name] experienced in a persistent or recurring way for more than 12 weeks since March 2020?

- Fever or high temperature
- Cough
- Lost or husky voice
- Chest pain or tightness
- Sore throat
- Feeling short of breath
- Chills or shivers
- Feeling lightheaded or disorientated
- Feeling dizzy
- Palpitations (feeling like your heart is beating heavily or racing)
- Weakness or tiredness
- Trouble sleeping
- Worry or anxiety
- Low mood or not enjoying anything
- Memory loss or confusion
- Difficulty concentrating
- Loss of smell or taste
- Headache
- Sore or uncomfortable eyes
- Earache or ringing in ears
- Stomach pain
- Diarrhoea
- Not feeling hungry or wanting to eat
- Feeling or being sick
- Raised, red, itchy bumps on skin or swelling of the face or lips
- Red or purple blisters on your feet or toes
- Prickling, tingling or burning sensations in skin
- Strong aches or pains in muscles or joints

## **Comparator definitions**

Comparisons in symptom prevalence were made between those reporting a history of a positive test and those with neither a positive test nor suspected COVID-19.

A positive test history was defined as a positive response to the question:

- Has [child’s first name] had a positive coronavirus (COVID-19) test of any kind since March 2020?

Suspected COVID-19 was defined as a positive response to the question:

- Do you know or think that [child’s first name] has had coronavirus (COVID-19) since March 2020?

# **Supplementary results**

# **Supplementary table 1** Prevalence of individual symptoms persisting for at least 12 weeks since March 2020 regardless of COVID-19 history, by age-group in survey rounds one and three

| Age-group | Symptoms | Weighted^1^ prevalence in round one (%, 95% C.I.) | Weighted^1^ prevalence in round three (%, 95% C.I.) |
| --- | --- | --- | --- |
| Primary school years 0-6 (aged 4 to 11 years) | Numbered symptoms  At least one  Three or more | 46.9 (44.3 to 49.5)  21.7 (19.6 to 23.9) | 48.0 (45.6 to 50.5)  23.8 (21.8 to 25.9) |
|  | Individual symptoms  Fever or high temperature  Cough  Lost or husky voice  Chest pain or tightness  Sore throat  Feeling short of breath  Chills or shivers  Feeling lightheaded or disorientated  Feeling dizzy  Palpitations  Weakness or tiredness  Trouble sleeping  Worry or anxiety  Low mood or not enjoying anything  Memory loss or confusion  Difficulty concentrating  Loss of smell or taste  Headache  Sore or uncomfortable eyes  Earache or ringing in ears  Stomach pain  Diarrhoea  Not feeling hungry nor wanting to eat  Feeling or being sick  Raised, red, itchy bumps on skin or swelling of the face or lips  Red or purple blisters on feet or toes  Prickling, tingling or burning sensations in skin  Strong aches or pains in muscles/ joints | 7.2 (6.0 to 8.7)  19.5 (17.5 to 21.7)  5.5 (4.4 to 6.8)  2.2 (1.5 to 3.1)  17.4 (15.5 to 19.5)  2.5 (1.8 to 3.5)  3.9 (3.0 to 5.0)  0.6 (0.3 to 1.2)  1.4 (0.9 to 2.2)  0.7 (0.3 to 1.3)  6.2 (5.0 to 7.6)  8.8 (7.4 to 10.4)  17.1 (15.2 to 19.2)  6.3 (5.1 to 7.7)  1.3 (0.7 to 2.0)  10.5 (9.0 to 12.2)  1.3 (0.8 to 2.1)  7.8 (6.5 to 9.3)  4.5 (3.5 to 5.8)  3.1 (2.2 to 4.1)  7.9 (6.5 to 9.4)  2.9 (2.1 to 3.9)  4.1 (3.1 to 5.2)  5.0 (3.9 to 6.2)  2.0 (1.4 to 2.9)  0.7 (0.3 to 1.2)  0.6 (0.3 to 1.2)  3.3 (2.4 to 4.3) | 8.0 (6.7 to 9.4)  17.6 (15.8 to 19.6)  5.2 (4.1 to 6.3)  2.9 (2.1 to 3.8)  13.0 (11.4 to 14.7)  4.1 (3.2 to 5.2)  5.3 (4.2 to 6.4)  1.9 (1.3 to 2.7)  2.9 (2.1 to 3.8)  1.4 (0.9 to 2.1)  9.8 (8.4 to 11.4)  10.1 (8.7 to 11.6)  18.5 (16.6 to 20.4)  8.9 (7.5 to 10.3)  1.5 (1.0 to 2.2)  10.7 (9.2 to 12.3)  2.0 (1.4 to 2.8)  9.9 (8.5 to 11.4)  5.7 (4.6 to 6.9)  3.6 (2.7 to 4.6)  10.4 (9.0 to 12.0)  4.0 (3.1 to 5.0)  5.4 (4.4 to 6.6)  6.7 (5.6 to 8.0)  2.2 (1.5 to 3.0)  0.5 (0.2 to 1.0)  0.9 (0.5 to 1.4)  4.4 (3.4 to 5.5) |
| Secondary school years 7-11 (aged 11 to 16 years) | Numbered symptoms  At least one  Three or more | 52.2 (48.3 to 56.0)  28.9 (25.5 to 32.4) | 52.9 (50.5 to 55.3)  30.0 (27.8 to 32.3) |
|  | Individual symptoms  Fever or high temperature  Cough  Lost or husky voice  Chest pain or tightness  Sore throat  Feeling short of breath  Chills or shivers  Feeling lightheaded or disorientated  Feeling dizzy  Palpitations  Weakness or tiredness  Trouble sleeping  Worry or anxiety  Low mood or not enjoying anything  Memory loss or confusion  Difficulty concentrating  Loss of smell or taste  Headache  Sore or uncomfortable eyes  Earache or ringing in ears  Stomach pain  Diarrhoea  Not feeling hungry nor wanting to eat  Feeling or being sick  Raised, red, itchy bumps on skin or swelling of the face or lips  Red or purple blisters on feet or toes  Prickling, tingling or burning sensations in skin  Strong aches or pains in muscles/ joints | 4.7 (3.2 to 6.5)  11.8 (9.5 to 14.4)  5.3 (3.7 to 7.2)  3.5 (2.3 to 5.2)  16.2 (13.6 to 19.2)  4.4 (3.0 to 6.2)  6.2 (4.5 to 8.2)  5.2 (3.6 to 7.1)  6.4 (4.6 to 8.5)  2.3 (1.3 to 3.7)  14.0 (11.5 to 16.8)  13.0 (10.5 to 15.7)  25.9 (22.6 to 29.4)  17.4 (14.6 to 20.4)  3.1 (1.9 to 4.7)  16.3 (13.6 to 19.3)  5.2 (3.7 to 7.1)  13.8 (11.3 to 16.6)  4.7 (3.3 to 6.6)  4.3 (2.9 to 6.1)  8.1 (6.2 to 10.5)  3.7 (2.4 to 5.4)  5.7 (4.1 to 7.7)  7.3 (5.5 to 9.5)  2.8 (1.7 to 4.3)  0.9 (0.4 to 2.0)  1.4 (0.6 to 2.6)  6.6 (4.9 to 8.8) | 6.1 (5.0 to 7.4)  12.0 (10.5 to 13.6)  5.6 (4.6 to 6.8)  4.8 (3.9 to 6.0)  15.6 (13.9 to 17.4) 6.4 (5.2 to 7.6)  5.9 (4.8 to 7.1)  6.4 (5.3 to 7.6)  7.4 (6.2 to 8.8)  4.3 (3.4 to 5.4)  16.7 (14.9 to 18.5)  13.5 (11.9 to 15.2)  25.2 (23.1 to 27.3)  17.8 (16.0 to 19.7)  3.2 (2.4 to 4.2)  16.3 (14.5 to 18.1)  7.0 (5.8 to 8.3)  13.3 (11.7 to 15.0)  4.9 (4.0 to 6.1)  3.5 (2.7 to 4.5)  8.5 (7.3 to 10.0)  3.6 (2.8 to 4.6)  7.5 (6.3 to 8.8)  7.1 (5.9 to 8.4)  2.3 (1.6 to 3.1)  1.1 (0.7 to 1.7)  1.6 (1.0 to 2.3)  7.6 (6.4 to 9.0) |
| Secondary school years 12 to 13 (aged 16 to 18 years) | Numbered symptoms  At least one  Three or more | 79.4 (68.7 to 87.7)  62.4 (50.7 to 73.1) | 79.5 (71.9 to 85.8)  68.1 (59.8 to 75.6) |
|  | Individual symptoms  Fever or high temperature  Cough  Lost or husky voice  Chest pain or tightness  Sore throat  Feeling short of breath  Chills or shivers  Feeling lightheaded or disorientated  Feeling dizzy  Palpitations  Weakness or tiredness  Trouble sleeping  Worry or anxiety  Low mood or not enjoying anything  Memory loss or confusion  Difficulty concentrating  Loss of smell or taste  Headache  Sore or uncomfortable eyes  Earache or ringing in ears  Stomach pain  Diarrhoea  Not feeling hungry nor wanting to eat  Feeling or being sick  Raised, red, itchy bumps on skin or swelling of the face or lips  Red or purple blisters on feet or toes  Prickling, tingling or burning sensations in skin  Strong aches or pains in muscles/ joints | 2.5 (0.3 to 8.9)  20.1 (11.9 to 30.7)  5.2 (1.5 to 12.7)  13.2 (6.6 to 22.7)  17.1 (9.6 to 27.3)  11.0 (5.0 to 20.1)  12.3 (5.9 to 21.7)  23.0 (14.2 to 33.9)  23.1 (14.4 to 34.0)  11.1 (5.1 to 20.3)  41.5 (30.5 to 53.2) 27.7 (18.1 to 38.9)  53.3 (41.7 to 64.7)  40.0 (29.1 to 51.7)  14.5 (7.6 to 24.3)  48.2 (36.8 to 59.8)  8.0 (3.0 to 16.3)  25.1 (16.0 to 36.2) 13.2 (6.6 to 22.8)  10.8 (4.9 to 19.9)  12.5 (6.1 to 22.0)  6.9 (2.4 to 14.9)  18.6 (10.7 to 29.0)  13.2 (6.6 to 22.8)  6.6 (2.2 to 14.5)  *  9.3 (3.9 to 18.0)  13.2 (6.6 to 22.8) | 8.9 (4.8 to 14.9)  19.5 (13.3 to 27.0)  12.8 (7.8 to 19.4)  16.4 (10.8 to 23.6)  22.6 (16.0 to 30.4)  21.0 (14.6 to 28.6)  20.0 (13.7 to 27.5)  28.1 (20.9 to 36.2)  25.0 (18.1 to 32.9)  18.1 (12.2 to 25.4)  52.3 (43.8 to 60.8)  35.1 (27.3 to 43.5)  54.1 (45.6 to 62.5)  51.4 (42.9 to 59.9)  22.4 (15.8 to 30.1)  48.4 (39.9 to 56.9)  11.4 (6.7 to 17.8)  29.5 (22.1 to 37.7)  23.4 (16.7 to 31.2)  18.3 (12.4 to 25.7)  16.5 (10.8 to 23.6)  5.1 (2.1 to 10.1)  25.7 (18.8 to 33.7)  13.6 (8.4 to 20.3)  12.4 (7.5 to 19.0)  3.6 (1.2 to 8.1)  4.5 (1.7 to 9.3)  19.7 (13.5 to 27.2) |

^1^ Estimates are weighted for sampling design, school-level response rate, school years, sex, ethnicity and free school meal eligibility (a measure of socio-economic deprivation) *suppressed due to small numbers

# **Supplementary table 2** Persisting symptom prevalence by COVID-19 status and age-group in survey rounds one and three

| Age-group | Symptom | Weighted^1^ prevalence in round one^*^, (%, 95% C.I.) | | | Weighted^1^ prevalence in round three^±^ (%, 95% C.I.) | | |
| --- | --- | --- | --- | --- | --- | --- | --- |
|  |  | Positive COVID-19 test since March 2020 | No reported COVID-19 since March 2020 | Difference | Positive COVID-19 test since March 2020 | No reported COVID-19 since March 2020 | Difference |
| Primary school years 0-6 (aged 4 to 11 years) | Numbered symptoms  At least one  Three or more | 47.5 (41.6 to 53.4)  26.0 (21.0 to 31.4) | 46.3 (43.3 to 49.4)  20.6 (18.2 to 23.1) | 1.2 (-5.5 to 7.8)  5.4 (-0.2 to 11.3) | 46.9 (43.6 to 50.2)  24.3 (21.5 to 27.2) | 49.3 (45.2 to 53.4)  23.6 (20.3 to 27.3) | -2.4 (-7.7 to 2.9)  0.7 (-3.9 to 5.1) |
|  | Grouped symptoms^∞^  Cardiovascular  Cognitive disturbance  Dermatologic  Gastrointestinal  HEENT  Loss of smell or taste  Mood  Musculoskeletal  Pulmonary  Systemic | 3.5 (1.7 to 6.3)  24.1 (19.3 to 29.5)  4.0 (2.1 to 7.0)  16.1 (12.1 to 20.9)  24.5 (19.7 to 29.9)  5.2 (2.9 to 8.4)  19.3 (14.9 to 24.3)  5.4 (3.1 to 8.7)  18.4 (14.1 to 23.4)  15.4 (11.5 to 20.1) | 2.2 (1.4 to 3.2)  19.5 (17.2 to 22.0)  2.7 (1.8 to 3.8)  11.7 (9.8 to 13.8)  20.2 (17.8 to 22.7)  [c]  17.7 (15.5 to 20.2)  2.5 (1.6 to 3.6)  20.3 (17.9 to 22.9)  10.2 (8.5 to 12.2) | 1.3 (-0.8 to 4.2)  4.6 (-0.8 to 10.5)  1.3 (-0.9 to 4.4)  4.4 (-0.1 to 9.6)  4.3 (-1.1 to 10.2)  [c]  1.6 (-3.5 to 7.1)  2.9 (0.4 to 6.3)  -1.9 (-6.9 to 3.6)  5.2 (0.8 to 10.2) | 4.8 (3.5 to 6.4)  23.6 (20.9 to 26.5)  3.7 (2.6 to 5.2)  15.8 (13.5 to 18.4)  18.5 (16.0 to 21.2)  3.1 (2.1 to 4.5)  20.6 (18.0 to 23.4)  5.1 (3.8 to 6.8)  17.2 (14.8 to 19.9)  15.3 (13.0 to 17.8) | 1.2 (0.5 to 2.4)  23.9 (20.6 to 27.6)  2.4 (1.3 to 4.0)  15.0 (12.2 to 18.1)  18.5 (15.5 to 21.9)  0.1 (0.0 to 0.9)  21.6 (18.3 to 25.1)  3.4 (2.1 to 5.2)  20.6 (17.4 to 24.0)  14.4 (11.7 to 17.5) | 3.6 (1.8 to 5.3)  -0.3 (-4.9 to 4.1)  1.3 (-0.6 to 3.2)  0.8 (-3.1 to 4.6)  0.0 (-4.2 to 4.0)  3.0 (1.7 to 4.4)  -1.0 (-5.4 to 3.3)  1.7 (-0.5 to 3.8)  -3.4 (-7.6 to 0.8)  0.9 (-3.0 to 4.6) |
| Secondary school years 7-11 (aged 11 to 16 years) | Numbered symptoms  At least one  Three or more | 57.6 (51.2 to 63.8)  32.5 (26.8 to 38.7) | 47.6 (42.7 to 52.6)  25.4 (21.3 to 29.9) | 10.0 (1.9 to 17.9)  7.1 (-0.2 to 14.5) | 55.4 (52.2 to 58.6)  31.4 (28.5 to 34.5) | 47.8 (43.9 to 51.6)  26.4 (23.1 to 30.0) | 7.6 (2.6 to 12.6)  5.0 (0.4 to 9.5) |
|  | Grouped symptoms^∞^  Cardiovascular  Cognitive disturbance  Dermatologic  Gastrointestinal  HEENT  Loss of smell or taste  Mood  Musculoskeletal  Pulmonary  Systemic | 3.4 (1.5 to 6.5)  31.5 (25.8 to 37.6)  6.4 (3.7 to 10.2)  17.7 (13.2 to 23.0)  21.6 (16.7 to 27.2)  16.6 (12.2 to 21.8)  23.6 (18.4 to 29.3)  9.6 (6.3 to 14.0)  16.6 (12.2 to 21.8)  22.2 (17.2 to 27.9) | 4.9 (3.0 to 7.4)  27.3 (23.1 to 31.9)  3.2 (1.7 to 5.4)  11.6 (8.7 to 15.2)  19.5 (15.8 to 23.7)  0.4 (0.0 to 1.6)  30.0 (25.6 to 34.7)  5.3 (3.4 to 8.0)  12.6 (9.5 to 16.2)  14.2 (11.0 to 17.9) | -1.5 (-4.6 to 2.1)  4.2 (-3.1 to 11.6)  3.2 (-0.3 to 7.3)  6.1 (0.3 to 12.1)  2.1 (-4.4 to 8.8)  16.2 (11.6 to 21.4)  -6.4 (-13.4 to 0.8)  4.3 (0.0 to 9.1)  4.0 (-1.7 to 10.1)  9.0 (1.8 to 14.5) | 8.5 (6.8 to 10.4)  32.2 (29.2 to 35.2)  3.6 (2.5 to 5.0)  14.5 (12.3 to 16.9)  20.3 (17.8 to 23.0)  10.3 (8.4 to 12.4)  30.1 (27.2 to 33.1)  8.7 (7.0 to 10.7)  16.0 (13.7 to 18.5)  22.1 (19.5 to 24.9) | 4.5 (3.1 to 6.4)  28.6 (25.2 to 32.2)  3.3 (2.1 to 4.9)  13.8 (11.3 to 16.7)  19.0 (16.1 to 22.2)  1.5 (0.7 to 2.8)  27.5 (24.1 to 31.1)  5.6 (4.0 to 7.7)  12.2 (9.8 to 14.9)  14.4 (11.8 to 17.3) | 4.0 (1.5 to 6.4)  3.6 (-1.1 to 8.1)  0.3 (-1.6 to 2.1)  0.7 (-2.9 to 4.2)  1.3 (-2.8 to 5.3)  8.8 (6.5 to 11.0)  2.6 (-2.0 to 7.1)  3.1 (0.4 to 5.7)  3.8 (0.3 to 7.3)  7.7 (3.8 to 11.5) |
| Secondary school years 12-13 (aged 16 to 18 years) | Numbered symptoms  At least one  Three or more | 92.0 (73.8 to 99.0)  82.2 (61.6 to 94.5) | 72.6 (57.5 to 84.7)  55.7 (40.3 to 70.3) | 19.4 (-2.5 to 36.0)  26.5 (1.3 to 46.2) | 88.9 (79.2 to 95.1)  73.6 (61.7 to 83.4) | 70.8 (58.5 to 81.2)  59.6 (47.0 to 71.3) | 18.1 (3.9 to 31.9)  14.0 (-2.7 to 30.0) |
|  | Grouped symptoms^∞^  Cardiovascular  Cognitive disturbance  Dermatologic  Gastrointestinal  HEENT  Loss of smell or taste  Mood  Musculoskeletal  Pulmonary  Systemic | 21.7 (7.8 to 42.8)  67.3 (45.6 to 84.6)  7.4 (0.8 to 25.4)  31.3 (14.3 to 52.9)  42.6 (23.1 to 63.9)  16.7 (4.8 to 37.1)  67.4 (45.7 to 84.7)  18.9 (6.1 to 39.6)  25.4 (10.2 to 46.8)  54.5 (33.4 to 74.5) | 18.3 (8.5 to 32.5)  59.3 (43.8 to 73.5)  16.0 (6.9 to 29.7)  28.5 (16.2 to 43.7)  35.5 (22.0 to 50.9)  [c]  56.6 (41.2 to 71.1)  13.0 (4.9 to 26.1)  27.2 (15.2 to 42.3)  38.9 (24.9 to 54.4) | 3.4 (-16.5 to 26.7)  8.0 (-17.9 to 31.2)  -8.6 (-23.8 to 11.6)  2.8 (-20.0 to 27.7)  7.1 (-17.7 to 32.3)  [c]  10.8 (-15.3 to 34.0)  5.9 (-12.4 to 28.1)  -1.8 (-23.2 to 22.7)  15.6 (-10.6 to 40.0) | 31.9 (21.3 to 44.1)  71.2 (59.2 to 81.4)  10.5 (4.5 to 20.1)  31.7 (21.2 to 43.9)  59.5 (47.2 to 71.0)  18.4 (10.2 to 29.4)  70.2 (58.1 to 80.5)  21.9 (12.9 to 33.4)  42.3 (30.6 to 54.7)  61.0 (48.7 to 72.4) | 17.2 (9.1 to 28.3)  63.4 (50.8 to 74.7)  19.4 (10.8 to 30.8)  34.0 (22.9 to 46.5)  39.6 (28.0 to 52.2)  1.3 (0.0 to 7.6)  58.1 (45.5 to 69.9)  13.3 (6.3 to 23.7)  19.0 (10.5 to 30.3)  44.6 (32.6 to 57.2) | 14.7 (-0.6 to 29.3)  7.8 (-8.7 to 24.0)  -8.9 (-21.8 to 4.0)  -2.3 (-18.6 to 14.2)  19.9 (2.3 to 36.2)  17.1 (6.8 to 28.2)  12.1 (-4.8 to 28.4)  8.6 (-5.2 to 22.1)  23.3 (7.0 to 38.3)  16.4 (-1.2 to 33.0) |

^1^ Estimates are weighted for sampling design, school-level response rate, school years, sex, ethnicity and free school meal eligibility (a measure of socio-economic deprivation)

^*^ In round one, 532 primary school pupils had a history of a positive test compared to 1,791 with no reported COVID. For years 7-11, 515 pupils reported a positive test history compared to 1,037 with no reported COVID. The corresponding figures for years 12-13 were 39 and 90.

^±^ In round three, 2,130 primary school pupils had a history of a positive test compared to 1,334 with no reported COVID. The corresponding figures were 2,027 and 1,415 for pupils in years 7-11 and 161 and 159 for pupils in years 12-13.

^∞^ Grouped symptoms comprised the following: cardiovascular (chest pain or tightness; palpitations), cognitive disturbance (feeling lightheaded or disorientated; feeling dizzy; trouble sleeping; headache; memory loss or confusion; difficulty concentrating), dermatologic (raised, red, itchy bumps on skin or swelling of face or lips; red or purple blisters on feet or toes; prickling, tingling or burning sensations in skin), gastrointestinal (diarrhoea; not feeling hungry or wanting to eat; feeing or being sick; stomach pain), HEENT (lost or husky voice; sore throat; sore or uncomfortable eyes; earache or ringing in ears), loss of smell or taste, mood (worry or anxiety; low mood or not enjoying anything), musculoskeletal (strong aches or pains in joints or muscles), pulmonary (cough; feeling short of breath), systemic (fever or high temperature; chills or shivers; weakness or tiredness).

[c] Suppressed due to small numbers
